# Current basic and preclinical research for treatment of radiation therapy–induced hyposalivation

**DOI:** 10.1016/j.jfscie.2025.100046

**Published:** 2025-04-16

**Authors:** Olga J. Baker, Harim Tavares dos Santos, Kihoon Nam

**Affiliations:** a Department of Otolaryngology-Head and Neck Surgery, University of Missouri, Columbia, MO;; b Department of Biochemistry, University of Missouri, Columbia, MO;; c Christopher S. Bond Life Sciences Center, University of Missouri, Columbia, MO;; d Department of Oral Biology, School of Dental Medicine, University at Buffalo, The State University of New York, Buffalo NY.

**Keywords:** Salivary glands, fibrosis, radiation, xerostomia, cancer

## Abstract

**Objectives.:**

Patients receiving radiation therapy (RT) for head and neck cancer experience hyposalivation, a condition that results in loss of oral health and significantly decreases the quality of life of millions of patients worldwide. Treatments include saliva substitutes and secretory agonists that provide only temporary relief and can result in significant adverse effects. To find more permanent and clinically viable solutions, alternative strategies are being developed that may restore salivary gland function in patients with head and neck cancer. This review provides an overview and basic interpretation of research for the treatment of RT-induced hyposalivation.

**Search Strategy.:**

The authors critique and synthesize a broad group of approaches that promote secretory function in an irradiated salivary gland.

**Citation Sources.:**

Three databases (PubMed, MEDLINE, Google Scholar) were searched for relevant peer-reviewed articles published from January 2000 through July 2024.

**Study Selection Criteria.:**

The authors selected research constituting a continuum from the most to least studied approaches to treating RT-induced hyposalivation with the goal of facilitating future work in the field.

**Data Elements Included.:**

The RT-induced hyposalivation treatments were categorized into post- and pre-RT headings, with approaches further distinguished according to their therapeutic goal, modality, and research stage, as well as with regard to their general pros and cons.

**Overall Conclusion.:**

A summary of the research approaches used to treat RT-induced hyposalivation has been created to encourage the development of improved treatments.

## Graphical Abstract

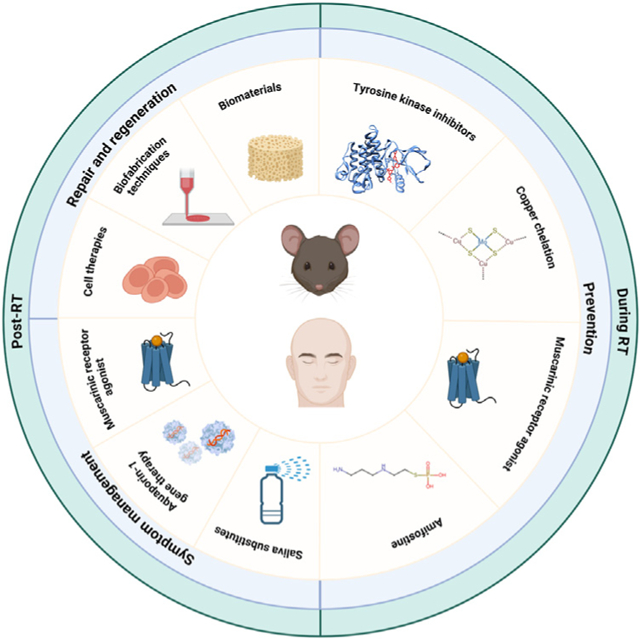

## Introduction

Head and neck cancer (HNC) is the seventh most common type of cancer, with more than 800,000 new cases annually worldwide.^1^ HNC remains challenging to treat, requiring a multidisciplinary approach, with radiation therapy (RT) being a key component of treatment, either to eliminate cancer or help control symptoms.^2^ However, RT leads to a loss of salivary gland (SG) function,^3^ as detailed below.

Hyposalivation, which refers to experimentally verifiable factors such as saliva flow rate (ie, < 0.1 mL/min), should not be confused with the related but more subjective term of xerostomia, which is the sensation of dry mouth typically present with hyposalivation.^4^ RT typically impairs SG function and leads to a progressive decrease in SG function because of apoptosis-driven parenchymal cell loss, inflammation, blood vessel dilation, function loss, nerve injury, reduced parasympathetic nervous function, and fibrosis.^5,6^

The psychosocial and emotional impact on the quality of life for patients with RT-induced hyposalivation is high as they experience loss of taste together with an inability to chew and swallow foods, all of which can result in serious nutritional deficiencies.^7^ Hyposalivation and consequent xerostomia also affect speaking and communication ability, and patients similarly experience nocturnal oral discomfort, thereby leading to sleep loss and additional generalized stress.^7^ Furthermore, with the prolonged oral clearance of sugars, the oral mucosa becomes painfully dry, sticky, and more susceptible to infection and the progression of caries as well as gingival and periodontal disease.^8^

Other risk-reducing interventions that may be offered during RT for HNC include bethanechol and acupuncture.^9^ The cited review also indicated that patients who develop SG hypofunction or xerostomia could be treated using topical mucosal lubricants, saliva substitutes, sugar-free lozenges, and chewing gum. Furthermore, oral pilocarpine and oral cevimeline, acupuncture, or transcutaneous electrostimulation may be offered after RT for patients with HNC.^9^ Although the approaches mentioned above are offering palliative results, continued research in this area is needed, and a host of approaches emerge each year attempting to address this issue.

The best approach to the problem of RT-induced SG damage among HNC patients would be to do less damage in the first place through using increasingly targeted RT. Although we would prefer to never damage the SG than to damage and later fix it, it is also true that RT is nowhere near being able to deliver on the ultimate goal of eliminating SG damage after radiation exposure while still delivering an effective treatment dose.^10–15^ Therefore, it is imperative that researchers and clinicians alike have a solid understanding of the range of treatment options for irradiated SG, and what follows is an attempt to summarize the field in an accessible manner.

## Methods

The scientific literature was searched to identify articles that contain research on hyposalivation with additional search criteria related to xerostomia, SG regeneration, biomaterials, cell therapies for restoring SG function, and approaches to protect SG from RT. Selected articles had to (1) fall within the January 2000 through July 2024 publication window, (2) denote the furthest research stage completed (eg, in vivo studies and clinical trials), (3) include at least 1 of the key words noted above in the body of the article, (4) have undergone a full peer review process terminating in formal publication (ie, no manuscripts indicated to be unpublished, submitted for publication, or under review) and (5) have an h-index of 30 or greater. Finally, we handsearched the titles and abstracts from those meeting the above inclusion criteria to verify that they did, in fact, provide information directly related to research for the treatment of RT-induced hyposalivation.

Regarding cell therapy studies, we conducted an advanced electronic search on the PubMed, MEDLINE and Google Scholar databases from January 2000 through July 2024. We defined a search strategy considering terms from the Medical Subject Headings, synonyms, and relevant terms on cell therapy and SGs combined with Boolean operators (ie, AND). We used no language or filter restrictions with the aim of generating a more comprehensive search. We then conducted the search using the following key words: inflammation, preclinical models, bioengineering, endogenous, cell transplantation, cell therapy, salivary gland, saliva, hyposalivation, xerostomia, radiotherapy, regeneration, acinar cells, fibrosis, stem cell, and mesenchymal stem cells (MSCs). EndNote Version 21 (Clarivate) was used as a software reference manager, and the selection process for articles considered consisted of 2 distinct phases performed by all 3 authors working together at all times. During the initial phase, only the titles and abstracts of the studies were reviewed. In the subsequent phase, we examined the full text of the articles. Any discrepancies were addressed through discussion until we reached a consensus. All preclinical and clinical studies that focused on the use of cell therapies to manage SG dysfunction induced by head and neck RT were included. Studies that did not address the use of cell therapies to treat SG dysfunction, in addition to duplicates, corrections, letters, and notes, were excluded.

## Results and Discussion

### Treatment strategies for RT-induced hyposalivation

The most commonly used and best explored broad treatment strategy to date is post-RT repair and regeneration, meaning that treatments are administered to either promote tissue growth or induce endogenous growth (eg, biomaterials and cell therapy).^16^ Because repair and regeneration, as evidenced by resolved inflammation and renewed saliva secretion, have not yet proven possible, viable posttreatment symptom management approaches have yet to be determined. Strategies in this category include attempts to induce some degree of saliva secretion or to repeatedly introduce a saliva substitute to reduce patient discomfort because of lost saliva secretion. Moreover, an emerging class of treatments seeks to prevent SG damage entirely by administering a protective treatment before RT. Finally, soluble bioactive factors are also core features of both regenerative medicine and tissue engineering and have thus been extensively reviewed elsewhere.^5,17^ In brief, a previous review^10^ reported the use of soluble bioactive factors that restore function in damaged SG, including insulinlike growth factor 1, the rapamycin analog CCI-779, and monoclonal antibodies that promote the ectodysplasin receptor signaling using a monoclonal antibody, whereas another review^11^ listed the use of additional soluble bioactive factors that promote SG tissue repair and function including intraductal infusion of interleukin 6 as well as studies involving both intraperitoneal injection with the P2X7R antagonist A-438079 as well as indomethacin. What follows is a brief explanation of the most prevalent or promising options in each category, which are summarized in the Table and Figure.

### Post-RT repair and regeneration

This area has the most coverage in our review because most of the research field has been devoted to biomaterial and cell therapy studies. In the past 15 years, material and cell-based approaches coupled with bioengineering have been used to advance studies on stimulating SG fluid secretion. Research has developed relevant animal models, dynamic tools, and reproducible methods to examine SG function reliably. Moreover, the SG research field has formed partnerships between salivary researchers with investigators from other disciplines, such as chemists, physiologists, radiation biologists, stem cell biologists, bioengineers, material scientists, and clinical investigators through the use of multiple principal investigator research project grants as well as an exploratory and developmental research grant format that has been encouraged by the National Institutes of Health’s National Institute of Dental Oral and Craniofacial Research in the United States.^18,19^ As a result, advances have occurred within the field of biomaterials- and cell-based SG bioengineering. These areas have evolved from concepts and ideas into practical tools for scientists and have become an integral part of many in vivo and clinical studies that provide insight toward solving clinical challenges.

#### Biomaterials

Biomaterials used in tissue engineering to restore function in RT-induced SG damage must meet some basic requirements. For instance, they should simulate SG function, provide a favorable environment for the formation of new tissue, and degrade once their role is fulfilled.^20^ To this end, they must have specific physical, mechanical, and chemical properties as well as biocompatibility. Below we describe major biomaterial-based approaches used to repair irradiated SG tissue.

##### Alginate-based hydrogels.

Alginate-based hydrogels are widely used in tissue engineering for their unique properties that mimic the natural extracellular matrix (ECM).^21^ These hydrogels are biocompatible, biodegradable, and capable of holding large amounts of water, creating a hydrated environment that supports cell survival and function.^22^ Alginate is nontoxic and has been shown to support the survival and proliferation of various cell types in vitro, including SG cells.^23^ This property makes it suitable for use in biomedical applications, including SG regeneration. Moreover, alginate-based hydrogels can hold large volumes of water, providing a moist environment that is crucial for maintaining cell viability and function. Likewise, this hydration capability mimics the natural ECM, supporting cell proliferation and differentiation. Alginate hydrogels encapsulate cells and bioactive molecules and can be injected, making them minimally invasive and suitable for delivering therapeutic agents directly to the target site. Similarly, a study using an adult mouse model showed that RT-damaged SG can be functionally regenerated via sustained delivery of the neurogenic muscarinic receptor agonist cevimeline.^24^ Specifically, endogenous SG repair coincided with increased nerve activity and acinar cell division that is limited to the first week after RT, with extensive acinar cell degeneration, dysfunction, and cholinergic denervation occurring thereafter.^24^ Researchers discovered that mimicking cholinergic muscarinic input via sustained local delivery of a cevimeline-alginate hydrogel is sufficient to regenerate innervated acini and retain physiological saliva secretion at nonirradiated levels over the long term in vivo (> 3 months).^24^ These studies represent an advancement in SG regeneration, and future clinical studies will be necessary to optimize their applicability to human participants.^21–24^

##### Hyaluronic acid (HA)-based hydrogels.

HA is a natural polysaccharide found in various connective tissues. It is known for its hydrophilic properties and ability to form a viscoelastic gel, which makes it an ideal candidate for use in tissue engineering and regenerative medicine.^25^ In the context of SG regeneration, HA-based hydrogels provide a supportive scaffold that mimics the natural ECM, facilitating the proliferation and organization of SG cells in vitro.^26^ The main benefits of using HA-based hydrogels include their biocompatible minimal immune response in vivo.^27^ In addition, HA-based hydrogels support the growth and proliferation of progenitor cells in vitro embryonic mouse submandibular glands (SMG) cell cultures, such as c-Kit^+^ cells, which are crucial for tissue regeneration.^26^ Moreover, the viscoelastic properties of HA-based hydrogels provide structural support for the formation of organized cell structures in vitro, such as acini and ducts, which are essential for SG function.^28,29^ Similarly, HA retains water and maintains a hydrated environment, which is beneficial for cell viability and function in vitro,^30^ and HA-based hydrogels may also be chemically and physically modified (eg, coimmobilization with polydopamine for better performance) to enhance their properties and tailor them for both in vitro and in vivo applications.^31^ The downside of HA-based hydrogels is their susceptibility to enzymatic degradation, which can limit the longevity of the hydrogel scaffold when applied in vivo, whereas the effectiveness of HA hydrogels can vary depending on their molecular weight and the method of presentation (solubilized vs immobilized), thereby requiring careful optimization for each specific application.^32^

##### Fibrin-based hydrogels (FHs).

The use of FHs in SG regeneration is based on their ability to create a supportive matrix that can promote cell growth, tissue organization, and regeneration. However, fibrin alone cannot replicate the complex 3-dimensional structure of the SG.^33^ To address this issue, FHs were fortified with laminin-1 peptides (L_1p_) (ie, A99 and YIGSR) to create a peptide-enhanced hydrogel (L_1p_-FH) in which rat parotid Par-C10 cells were cultured in vitro.^33^ The addition of L_1p_ improved the bioactivity of the fibrin matrix by promoting cell adhesion, migration, and organization, all of which are crucial for SG tissue regeneration. The major benefits of L_1p_-FH included enhanced epithelial tissue organization and improved cell survival rates; however, this scaffold lacked the necessary growth factors for supporting angiogenesis and neurogenesis, both of which are essential for the structural and functional recovery of the SG in vivo.^34^ To overcome these limitations, an enhanced FH (Ep-FH) was developed by fortifying L_1p_-FH with growth factors (FGF-7 and FGF-10).^35^ This new Ep-FH scaffold involves the promotion of epithelial tissue organization as well as angiogenesis and neurogenesis, all of which are essential for restoring the SG function of irradiated mouse SG. Ep-FH elicits its effects over an extended period (ie, ≈90 days in a mouse lifetime), suggesting long-term benefits in vivo. Although Ep-FH represents an alternative to restore SG function, future studies will be necessary to identify its mechanism and apply it to humanized mouse models before this therapy can be used in the clinic.

##### Poly(lactic-glycolic acid) (PLGa)-based scaffolds.

PLGa-based scaffolds are designed to mimic the ECM, providing a supportive environment for the attachment, proliferation, and differentiation of cells and ex vivo SMG organ cultures.^36^ These scaffolds help maintain SG epithelial integrity in regenerated tissue while facilitating the proper organization of cells into functional glandular units. Moreover, PLGa-based nanoparticles and microspheres have been used to deliver growth factors and bioactive molecules in a targeted manner to repair SG damage, thereby enhancing the regeneration process through the promotion of cell proliferation and differentiation.^37^ In addition, the release of therapeutic agents from PLGa-based delivery systems ensures a sustained effect, improving the overall efficacy of the treatment to restore SG function in vivo (eg, in rat parotid glands and ICR strain irradiated mice).^38,39^ The major drawbacks of PLGa-based hydrogels includes the potential for local inflammation caused by the byproducts of PLGa degradation and decreased stability of encapsulated drugs^40^ and production requires precise control of parameters to achieve desired properties, which can be technically challenging.

##### Polyethylene glycol (PEG)-based hydrogels.

PEG-based hydrogels serve as scaffolds that provide a 3-dimensional structure, thereby supporting the growth and differentiation of primary mouse SMG cells in vitro.^41^ PEG-based hydrogels are highly biocompatible and can be modified to include specific peptides or growth factors that enhance cellular interactions in vitro.^42–44^ Moreover, PEG can be used to encapsulate and deliver growth factors, cytokines, and other therapeutic agents^45^ directly to the damaged SG tissue. The controlled release properties of PEG hydrogels ensure sustained delivery of these agents, enhancing the regenerative process. Furthermore, PEG improves the stability and solubility of encapsulated drugs in vitro,^46^ ensuring their effective delivery and activity at the SG. PEG hydrogels can also encapsulate stem cells, which in turn promotes acinar cell viability and functionality in vitro,^47^ a step that may be considered crucial for improving their engraftment and integration into the damaged SG tissue in vivo. Finally, the mechanical and chemical properties of PEG-based hydrogels can be tailored to match the specific requirements of the SG tissue, thus promoting optimal conditions for cell differentiation and tissue regeneration.^48^ The major drawback of PEG-based hydrogels is their rapid degradation in vivo.^49^ Specifically, although PEG is generally considered biocompatible, controlling the degradation rate can be challenging and may affect the release profile of encapsulated drugs.

##### Decellularized ECM (DECM) hydrogels.

The injectable DECM hydrogel, derived from decellularized porcine SMG, was designed to promote SG regeneration by providing a biocompatible scaffold that mimics the native ECM in injured rat SMG in vivo.^50^ Moreover, DECM facilitates the migration and recruitment of SG-derived MSCs, supports tissue regeneration, and suppresses fibrogenesis through activation of the phosphatidylinositol 3-kinase and protein kinase B signaling pathway. Furthermore, it is injectable and forms in situ at body temperature, thereby allowing for minimally invasive administration and filling of irregular defects. The main issue with DECM is its high variability between batches because of immunologic differences in donor tissues, which leads to inconsistencies in the composition and properties of the DECM^51^; as a result of these collection-related complications, further studies will be needed to confirm its safety for use in humans by developing standards for adequate sample evaluation.

##### Gelatin-based hydrogels.

Gelatin-based hydrogels can be manufactured in various forms and tailored to specific regenerative needs.^52^ The properties of gelatin-based hydrogels are adjustable through photopolymerization processes to control mechanical strength and degradation rates. Moreover, they promote cell adhesion and proliferation, facilitating SG regeneration in resected rat SMG in vivo.^53^ However, gelatin-based hydrogels degrade relatively quickly in vivo, thereby rendering them unsuitable for long-term applications.

#### Biofabrication Techniques

Biofabrication techniques are manufacturing approaches that combine biological components with a matrix or substrate to create biological products, thereby offering the potential to mimic the structural and functional complexity of living tissues successfully.^54^ To this end, biofabrication techniques represent a critical shift toward more controlled and replicable tissue engineering processes, thereby enhancing the efficacy and success rate of regenerative therapies for SG damage caused by RT. Common biofabrication techniques used to repair damaged SG are detailed below.

##### Bioprinting.

This technique allows for precise placement of cells and biomaterials, creating complex tissue structures that closely resemble natural tissues. Bioprinting has been used to produce multilayered scaffolds that support varied cellular environments, a step that is essential for the functional restoration of irradiated SG tissues. Advanced bioprinting techniques have likewise showed the potential for fabricating highly organized SG structures in vitro.^55,56^ However, the translation of these techniques from in vitro models to in vivo applications remains a considerable challenge and an area of active research. Consequently, further studies are needed to assess the viability and functionality of these bioprinted tissues in living organisms.

##### Electrospinning.

Producing ultrafine fibers through electrospinning results in scaffolds that are functionally analogous to the ECM, thereby enhancing cellular attachment and proliferation. These fibrous scaffolds provide excellent porosity and surface area, which are beneficial for the integration and function of SG cells.^57,58^ Moreover, studies have shown that electrospun scaffolds can be enhanced with bioactive molecules to increase their regenerative properties.^57,58^ For instance, elastin-based nanofibers have been developed to provide biomechanical and biochemical cues that closely mimic those of the natural SG environment, thereby promoting the organization and functional recovery of SG cells. These scaffolds support the growth and differentiation of various cell types, essential for effective tissue regeneration. The integration of these advanced materials within electrospun scaffolds addresses the complex requirements of SG tissue engineering, such as the need for precise cell placement and the formation of structured, lumenized tissue architectures essential for restoring SG functionality. Nonetheless, challenges remain in achieving uniform cell distribution throughout these scaffolds and in matching the mechanical properties to those of the native tissues, factors that are critical for the functional integration of the regenerated glands.

##### Thermal molding.

This method involves the shaping of polymers using heat and pressure, thereby creating scaffolds with glandular shape and characteristics.^48,59^ Thermal molding is particularly effective for producing robust, biodegradable scaffolds that maintain structural integrity under physiological conditions, which is crucial for long-term implantation.^48,59^ These scaffolds can be readily formed into coversliplike disks that are suitable for cell seeding; however, although these scaffolds facilitate the initial cell placement, they often require additional ECM components to enhance cell attachment and proliferation. Therefore, additional studies are needed to address these bioactivity issues that are essential for successful tissue regeneration.^48,60^

##### Freeze-drying.

Freeze-drying is used to create porous scaffolds that facilitate nutrient and oxygen diffusion to support the formation of vascularized tissue, factors that are essential for cell survival and tissue maturation in SG tissue engineering.^20,61^ The particular advantage of this technique is that it provides topographic cues to promote epithelial cell growth and facilitate the secretion of ECM proteins while retaining differentiated function.^20^ However, these scaffolds often require a fibronectin coating to enhance cellular interaction, and although they effectively mimic the basement membrane for epithelial cells, they may not be ideal for stromal cell culture because of a lack of in vivo-like viscoelastic properties.^62,63^ Therefore, additional studies to improve their mechanical properties are needed to make them suitable for clinical applications.

#### Cell Therapies

Similar to biomaterials, cell-based therapies are widely used to repair irradiated SG tissue. An important factor that ensures the safety and effectiveness of cell-based therapies is the careful selection of high-grade raw materials. Moreover, the transplanted cells must be functional and compatible with the recipient-irradiated SG. Finally, the cells used for these regeneration therapies must be consistent and reproducible to ensure reliable and predictable results. Below are described major cell-based therapies that have been used to repair irradiated SG tissue.

##### Kit^+^ cells.

This cell-based therapy involves isolating cells that express the cell surface receptor Kit, followed by the autologous transplantation of Kit^+^ cells to irradiated mouse SG in vivo.^64–66^ The main benefit of this approach is that Kit^+^ cells promote the formation of SG structures, angiogenesis, reduction of fibrosis, and increased saliva flow rates in irradiated SG.^64–66^ However, a major issue with this approach is the progressive depletion of stem and progenitor cells in aging patients, which may decrease the availability of Kit^+^ cells, thereby limiting autologous transplantation in some patients. Another limitation is the short lifespan of Kit^+^ cells in culture and their tumorigenic potential, posing a health risk for this type of therapy.

##### Bone marrow-derived MSCs (BM-MSC).

Researchers obtained BM-MSC from mouse tibias or femurs and applied them to treat RT-induced SG damage.^67–71^ The main benefits of this approach are the preservation of acinar and ductal structures, promotion of angiogenesis, and increased saliva flow rates in irradiated mice and rat SG in vivo.^67–71^ However, the laborious process of harvesting bone marrow cells and the unclear number of cells needed for successful regeneration are major limitations.^69^ In addition, the underlying mechanisms by which BM-MSCs repair irradiated SG damage are still poorly understood,^71^ and there is likewise controversy over whether BM-MSCs can actually differentiate into SG cells.^72^ Another approach involves delivering interferon-gamma–activated BM-MSCs obtained from the posterior superior iliac crest for the treatment of xerostomia in patients who have had no evidence of HNC for 2 or more years after completing RT. Specifically, a study protocol for a phase I dose-escalation trial of patients with xerostomia after RT HNC proposed injection of BM-MSCs into SMG using ultrasound guidance, with dental health and saliva quantity and quality to be assessed for 24 months^73^; however, major limitations of this protocol include the lack of placebo control, a small number of participants and no histologic analyses.

##### Adipose tissue-derived MSCs (ADSC).

Researchers obtained ADSC from inguinal fat pads and transplanted them into irradiated mice, rats, and minipigs SG in previous in vivo studies.^74^ This treatment increased SG weight,^74^ improved SG angiogenesis, reduced SG cell apoptosis,^75^ and decreased collagen deposition.^76^ Moreover, ADSC led to higher mucin and amylase production levels compared with irradiated SG controls^74,76–78^ while also improving saliva flow rates^74,77–81^; however, a major limitation of this approach is a lack of time-dependent studies to determine the optimal time of application to treat irradiated SG damage in vivo.^75^ Furthermore, the mechanisms by which ADSC protects SG against RT-induced damage remain unclear and the long-term effects of ADSC for promoting irradiated SG repair are yet to be determined. A similar study using human participants expanded and purified ADSC in vitro for 14 days before being injected intraglandular and guided by ultrasound into patients with post-RT-induced xerostomia.^82^ This treatment was observed to increase acinar and ductal cell areas and saliva flow rates for up to 4 months after treatment and showed lower collagen deposition together with a reduction of xerostomia compared with control participants^82,83^; however, limitations of this approach include no histologic analysis and the small sample size, so future randomized controlled trials will be necessary for using this approach to be reliably applied in the clinic.^83^ Moreover, a phase II clinical trial showed that ADSC treatment led to a noticeable improvement in unstimulated salivary flow compared with the placebo, although the difference between the groups was not statistically significant. Specifically, both ADSC and placebo groups experienced reductions in dry mouth symptoms and swallowing difficulties, but no significant differences were found in these 2 groups.^84^ Although these findings cannot establish clinical relevance because of the absence of statistical significance at the levels measured within the studies cited, they nonetheless provide suggestions for future directions to study such that ADSC may be determined to increase the saliva flow rate.

##### Effective mononuclear cells.

This therapy is based on in vitro manipulation of peripheral blood mononuclear cells using vasculogenic proteins, resulting in an enriched population of cluster of differentiation 11b and cluster of differentiation 206-positive (M2 macrophagelike) cells, named effective conditioned mononuclear cells (E-MNC).^85^ The benefit of this approach is the promotion of angiogenesis, increased SG differentiation, and recovery of secretory function in an irradiated mouse model. However, a major risk of using this approach is the possible activation of tumor proliferation, angiogenesis, or metastasis because of the vasculogenic properties of E-MNCs.^85^ Moreover, E-MNCs have been used clinically and transplanted into the SMG of human participants with RT-induced xerostomia.^86^ An advantage of E-MNC is the minimally invasive process required to obtain peripheral blood mononuclear cells compared with other autologous therapeutic cells such as ADSC.^86^ To this end, a phase I clinical trial delivered E-MNC directly into SMG using ultrasound-guided injections in patients who had no recurrence of HNC for more than 5 years after RT and were experiencing xerostomia.^86^ Preliminary data indicate that although this treatment increased saliva production, it did not cause irreversible adverse effects in humans.^86^ However, similar to their use in preclinical studies, this cell-based therapy carries the risk of inducing tumor growth and metastasis.^86^

##### SG stem cells (SGSC).

A study has established an SG stem cell clone isolated from human parotid glands using a subfractionation culturing method for the selection of homogenous clonal stem cells.^58^ These cells display stem cell features, including self-renewal, trilineage differentiation, and sphere-forming ability. Transplantation of SGSCs into irradiated mouse SMG resulted in increased mucin expression, preservation of acinar and ductal cells, decreased levels of both DNA damage and apoptosis as well as restored saliva flow rates compared with healthy control participants. However, concerns have been raised regarding the translational relevance of the in vivo model, thus indicating that the signaling mechanisms of this radioprotective cell therapy are still poorly understood.^58^ Furthermore, a 2024 study showed the successful optimization and production of SGSCs within a good manufacturing practice process framework.^87^ Specifically, this study produced clinical-grade batches of SGSC that retained the capability to self-renew, differentiate, and function effectively, results that have led to an ongoing phase I clinical trial to treat SG dysfunction.^87^

##### SG cells derived from embryonic stem cells (ESCs).

Previous studies have isolated mouse early ESCs from the inner cell mass of blastocysts.^88,89^ Moreover, a 2013 SG-oriented study indicated that mouse early ESCs can differentiate into SG cells when cocultured with human SG-derived fibroblasts in vitro.^89^ The same group likewise observed that when ESC-derived SG cells were transplanted into healthy mouse SMG in vivo, these cells could enhance acinar and ductal formation, thereby indicating that the transplantation of ESC-derived SG cells induces regeneration of neo-SG tissues.^89^ Finally, SG regeneration can only occur if some portion of the recipient SG is functional, making it unsuitable for participants with full fibrotic tissue coverage.^89^ Therefore, future studies in this area should seek to use regenerative methods in instances in which no healthy native tissue can be used as a basis for further growth.

### Post-RT symptom management

This area is a middle ground of sorts, offering both broad research and even broader clinical applications. Although the approaches contained herein do not solve the problem of RT-induced hyposalivation and, in fact, tend toward incomplete and impermanent effects, it is undeniable that this class of strategies is beneficial to the patient because relief is at hand because of their ready availability. Below is a brief sampling of such clinically available approaches presented not as a complete list but rather as an overview of possibilities in this area with a discussion of both their strengths and limitations.

#### Muscarinic Receptor Agonists

Pilocarpine and cevimeline are muscarinic receptor agonists that induce saliva secretion from residual acinar cells^90,91^; however, their use provides only temporary relief, requires some degree of intact SG tissue, and involves significant adverse effects.^92,93^ In addition, muscarinic agonists in combination with methacholine have been used during RT, with clinical results indicating that this combination also reduces radiation-induced SG damage.^94^ Muscarinic receptor agonists (eg, bethanechol and pilocarpine) have also been studied in relation to RT prevention, both in animals and humans (see below).

#### AQP1 *Gene Therapy*

Previous studies showed that adenovirus-mediated *hAQP1* gene delivery restores saliva secretion in head and neck irradiated in vivo animal models, including mice, rats, and minipigs.^95,96^ These studies were followed by a phase I clinical trial in which a recombinant serotype 5 adenoviral vectors encoding hAQP1 was applied via retroductal delivery to the parotid glands, causing an improvement of saliva secretion in 6 of 11 patients experiencing hyposalivation because of head and neck RT.^97^ Patients responding to the treatment displayed modest immune reactivity after gene transfer compared with the nonresponders. However, this study would need to be corroborated with a larger population. Subsequent work used another adeno-associated virus (AAV2)-mediated *hAQP1* gene delivery to irradiated minipig parotid glands, which also restored saliva secretion while inducing long-term gene expression in mouse parotid glands without significant adverse effects.^98^ As AAV2-based vectors exhibit lower immunogenicity and more stable expression than adenoviral vectors, they appear to be a more promising gene therapy alternative for hyposalivation. A phase I clinical trial is testing the safety of a single administration of AAV2*hAQP1* to 1 parotid SG in patients with RT-induced hyposalivation.^99^ Nevertheless, the delivery of the *AQP1* gene continues to be a promising treatment pending the successful completion of clinical trials. Finally, safer gene delivery methods using nontoxic biomaterials or nonviral methods are needed to improve the safety of this treatment.

#### Saliva Substitutes

Although a host of products emerge on the market each year attempting to address the loss of saliva, all to date lack the critical properties of saliva and thus fail to provide the desired relief.^100^ By way of defining the problem, we believe that 3 core factors are required for a saliva substitute to bring relief to patients truly. First, the mouthfeel is critical,^101^ meaning that it must be tolerated and remain within the mouth for an extended time (ie, viscoelasticity and mucoadhesion, respectively). Next, the saliva substitute must aid in digestion^102^ as well as both including neutral fluoride preparations as well as maintaining proper pH within the oral cavity, both of which are important to reduce the risk of caries.^103,104^ It is our opinion that none of the options on the market truly satisfy these criteria, most notably the first, primarily because of a lack of ability to include mucins, which are the core component of natural saliva, at an affordable price and with a sufficient degree of purity.^103,105^

### During RT prevention

This area is also brief and not inclusive of all innovative strategies for preventing RT hyposalivation.^9^ However, what follows is an attempt to document first the most clinically viable approach, then the most comprehensive preventative approach (ie, covering both protection and repair of damaged tissue), and finally, to offer an example of speculative work that might take the field in previously unconsidered directions. In so doing, we aim to present examples of what may be possible in what is, to date, a poorly documented area of research on the problem of RT-induced hyposalivation.

#### Amifostine

Amifostine is a radioprotective US Food and Drug Administration–approved drug used to reduce the occurrence of xerostomia in patients with HNC by way of providing a reduction of RT-related damage to SG parenchyma.^106^ The effects of amifostine have been extensively reviewed elsewhere.^9^ Briefly, a clinical study shows that amifostine works, although the effect is limited and the adverse effects (eg, hypotension and nausea) are major with respect to the potential clinical gain.^9^ Regarding preclinical studies, findings show that retroductal delivery of amifostine to mouse SMG before a single radiation dose of 15 Gy maintained SG function and significantly increased acinar cell survival.^9,107^ Furthermore, in vivo stimulated saliva secretion was maintained in retroductal delivery-treated groups at levels significantly higher than irradiated-only and systemically treated groups.

#### Tyrosine Kinase Inhibitors (TKIs)

Previous studies showed that TKIs (eg, dasatinib and imatinib) provide robust and durable radioprotection of SG function and that imatinib promotes SG regeneration after RT both in vitro and in vivo.^108,109^ A follow-up study identified a role for TKI in regulating DNA repair and showed that extracellular signal-regulated kinase activation plays a role in TKI-mediated cell survival and cell proliferation.^109^ Resultant increased repair of DNA damage, resistance to apoptosis, and enhanced proliferation are all reliable contributors to the preservation of SG function in vivo.^109^ Although these findings support the use of TKI as a therapeutic strategy for the protection of nontumor SG tissue in patients undergoing HNC therapy, future clinical studies will be necessary to confirm that TKI functions effectively in human tissues.

#### Muscarinic Receptor Agonists

Although muscarinic agonists have been used in post-RT symptom management, they have also been studied with regard to RT prevention, both in animals and humans. Regarding animal studies, it was shown that long-term administration of pilocarpine has beneficial effects on salivary flow in irradiated mice, and results likewise suggested that long-term administration may decrease apoptosis in irradiated SG.^110,111^ Regarding humans, a 2023 systematic review and meta-analysis indicate that bethanechol can help reduce the incidence of xerostomia and hyposalivation throughout RT in patients with HNC.^112^ Similarly, an earlier meta-analysis showed that concomitant administration of pilocarpine during radiation could increase the unstimulated salivary flow rate and reduce clinician-rated xerostomia grade.^113^ Results further suggest that it may also relieve patients’ xerostomia for up to 12 months; however, no effects on stimulated salivary flow rate were observed.^113^ Together, these meta-analyses indicate the prophylactic administration of muscarinic agonists during RT may reduce damage in irradiated SG.^112,113^

#### Regulating Copper (Cu) Availability via Metal Chelation

As stated above, RT can lead to fibrosis of the SG and hyposalivation.^5^ All the therapeutic strategies to restore SG function listed above depend on the presence of residual functional SG tissue, a condition not met with full RT because of extensive fibrotic coverage of the SG. Although the role of lysyl oxidase (LOX) in RT-induced fibrosis has not been investigated in SG, each LOX family member has been implicated in various fibrotic disorders in multiple organs.^114,115^ This led to the generation of selective monoclonal antibodies targeting LOX enzymes. However, phase II clinical trials using antibodies against LOX-like failed to show efficacy against primary sclerosing cholangitis (a fibrotic disease).^116^ These findings highlight the importance of considering functional redundancy when targeting individual LOX. Because the catalytic site of all LOX family members possesses a conserved binding site for Cu, focusing on the inhibition of Cu delivery to block these enzymes is a novel antifibrotic strategy.^117^ To this end, a study showed that transdermal administration of the Cu chelator tetrathiomolybdate (TTM) into mouse SMG reduced early collagen deposition and preserved saliva secretion in a neck RT mouse model.^118^ Mechanisms of this process may best be understood in relation to Wilson disease, a condition characterized by Cu overload that is managed by reducing bioavailable Cu using TTM which binds with high affinity and specificity to Cu ions. TTM has proven efficacy in preclinical models for fibrosis in multiple tissues; however, its application to RT-induced SG damage was previously untested. This study represents a new approach to the untreatable problem of RT-induced SG fibrosis.^118^ More work is needed to understand the downstream signaling mechanism of Cu chelation on SG fibrosis and the long-term effects of TTM on collagen deposition.

## Conclusions

The overall narrative pursued in our review, involving a move from the most to least studied approaches, is intended as a structure for facilitating future work. The best-understood approaches, broadly described as including both the study of a host of biomaterials and cell therapies, still require investigation of their mechanisms to be pushed through to the clinically viable stages. Moreover, the clinically available treatments, those that we have categorized as symptom management, should not be overlooked. Although they offer only partial and temporary effects, these treatments are all the patients can count on and will no doubt continue to play a significant role even as the repair and regeneration approaches are improved or preventative options emerge as practical alternatives, given that residual symptoms can always be counted on to emerge and should thus be planned for. In addition, prevention is a difficult area to pursue because causes are generally poorly understood, and early identification methods are lagging in development; however, this should not deter us from trying to get to the root of the problem and keep it from manifesting. We hope that this overview helps researchers and clinicians alike to better understand research performed to date and that it may even encourage some to pursue the development of improved treatments for RT-induced hyposalivation.

## Figures and Tables

**Figure:**
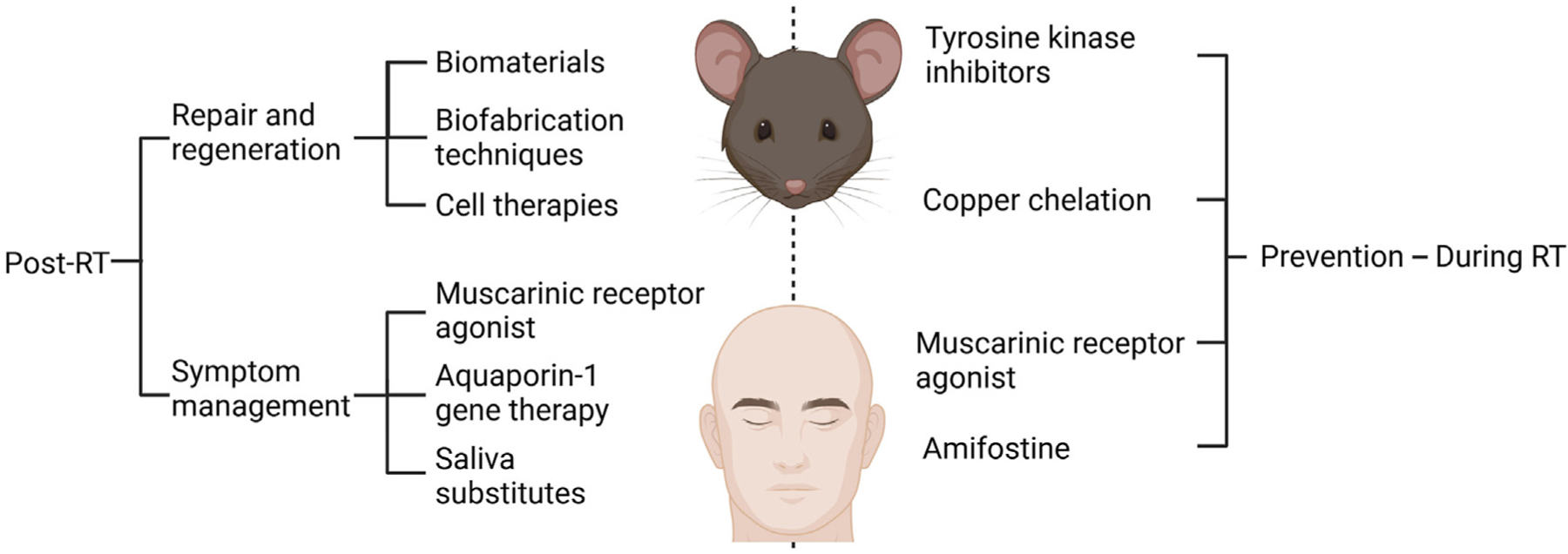
Summary of approaches for treating radiation-induced salivary gland hypofunction created in BioRender.com. RT: Radiation therapy.

**Table:** RT*-induced hyposalivation research overview.

Timing	Goal	Modality	Research Stage	Pros	Cons
Post-RT^†^	Repair and regeneration	Biomaterials	Validated in vivo, mechanisms not identified	Produced in quantity	Overly simplistic
		Biofabrication techniques	Validated in vitro	Support cell growth in vitro	Lacks in vivo validation
		Cell therapies	Validated in vivo, mechanisms not identified	Comprehensive and autologous	Hard to scale
	Symptom management	Muscarinic receptor agonist	Clinical	Noninvasive with rapid effects	Incomplete and impermanent
		Aquaporin-1 gene therapy	Clinical		
		Saliva substitutes	Clinical		
During RT	Prevention	Amifostine	Clinical	US Food and Drug Administration-approved and contributes to protection	Partial fix with severe adverse effects
		Tyrosine kinase inhibitors	Validated in vivo, mechanisms partially identified	Contributes to protection and repair	Partial fix with significant adverse effects
		Muscarinic receptor agonist	Preclinical and clinical	Reduces xerostomia and hyposalivation	Unclear long-term effectiveness
		Copper chelation	Proof of concept in vivo, largely speculative with unknown mechanisms	Could solve the problem	Early stages

* RT: Radiation therapy.

† Post-RT repair and regeneration is the most researched area, whereas post-RT symptom management represents the most clinically viable body of approaches, and pre-RT prevention offers the greatest potential upside.

## References

[1] Mody MD, Rocco JW, Yom SS, Haddad RI, Saba NF. Head and neck cancer. Lancet. 2021;398(10318):2289–2299. doi: 10.1016/S0140-6736(21)01550-6 34562395

[2] Guan Z, Liu J, Zheng L. Effect of radiotherapy on head and neck cancer tissues in patients receiving radiotherapy: a bioinformatics analysis-based study. Sci Rep. 2024;14(1):6304. doi: 10.1038/s41598-024-56753-4 38491080 PMC10943217

[3] Hong C, Jensen SB, Vissink A, MASCC/ISOO clinical practice statement: management of salivary gland hypofunction and xerostomia in cancer patients. Support Care Cancer. 2024;32(8):548. doi: 10.1007/s00520-024-08688-9 39048728 PMC11550240

[4] Lin A, Helgeson ES, Treister NS, The impact of head and neck radiotherapy on salivary flow and quality of life: results of the ORARAD study. Oral Oncol. 2022;127:105783. doi: 10.1016/j.oraloncology.2022.105783 35231809 PMC8977268

[5] Jasmer KJ, Gilman KE, Muñoz Forti K, Weisman GA, Limesand KH. Radiation-induced salivary gland dysfunction: mechanisms, therapeutics and future directions. J Clin Med. 2020;9(12):4095. doi: 10.3390/jcm9124095 33353023 PMC7767137

[6] Liu Z, Dong L, Zheng Z, Mechanism, prevention, and treatment of radiation-induced salivary gland injury related to oxidative stress. Antioxidants (Basel). 2021;10(11):1666. doi: 10.3390/antiox10111666 34829539 PMC8614677

[7] Patton LL, Helgeson ES, Brennan MT, Oral health-related quality of life after radiation therapy for head and neck cancer: the OraRad study. Support Care Cancer. 2023;31(5):286. doi: 10.1007/s00520-023-07750-2 37079106 PMC11216743

[8] Flink H, Tegelberg Å, Arnetz JE, Birkhed D. Self-reported oral and general health related to xerostomia, hyposalivation, and quality of life among caries active younger adults. Acta Odontol Scand. 2020;78(3):229–235. doi: 10.1080/00016357.2019.1690677 31729277

[9] Mercadante V, Jensen SB, Smith DK, Salivary gland hypofunction and/or xerostomia induced by nonsurgical cancer therapies: ISOO/MASCC/ASCO guideline. J Clin Oncol. 2021;39(25):2825–2843. doi: 10.1200/JCO.21.01208 34283635

[10] Lan X, Chan JYK, Pu JJ, Saliva electrolyte analysis and xerostomia-related quality of life in nasopharyngeal carcinoma patients following intensity-modulated radiation therapy. Radiother Oncol. 2020;150:97–103. doi: 10.1016/j.radonc.2020.06.016 32544605

[11] Taylor A, Powell ME. Intensity-modulated radiotherapy: what is it? Cancer Imaging. 2004;4(2):68–73. doi: 10.1102/1470-7330.2004.0003 18250011 PMC1434586

[12] Vergeer MR, Doornaert PAH, Rietveld DHF, Leemans CR, Slotman BJ, Langendijk JA. Intensity-modulated radiotherapy reduces radiation-induced morbidity and improves health-related quality of life: results of a nonrandomized prospective study using a standardized follow-up program. Int J Radiat Oncol Biol Phys. 2009;74(1):1–8. doi: 10.1016/j.ijrobp.2008.07.059 19111400

[13] Nutting CM, Morden JP, Harrington KJ, ; on behalf of the PAR-SPORT trial management group. Parotid-sparing intensity modulated versus conventional radiotherapy in head and neck cancer (PAR-SPORT): a phase 3 multicentre randomised controlled trial. Lancet Oncol. 2011;12(2):127–136. doi: 10.1016/S1470-2045(10)70290-4 21236730 PMC3033533

[14] Steenbakkers RJHM, van Rijn-Dekker MI, Stokman MA, Parotid gland stem cell sparing radiation therapy for patients with head and neck cancer: a double-blind randomized controlled trial. Int J Radiat Oncol Biol Phys. 2022;112(2):306–316. doi: 10.1016/j.ijrobp.2021.09.023 34563635

[15] van Rijn-Dekker MI, la Bastide-van Gemert S, Stokman MA, Radiation-induced xerostomia is related to stem cell dose-dependent reduction of saliva production. Int J Radiat Oncol Biol Phys. 2024;120(3):772–782. doi: 10.1016/j.ijrobp.2024.04.012 38631537

[16] Haubner F, Ohmann E, Pohl F, Strutz J, Gassner HG. Wound healing after radiation therapy: review of the literature. Radiat Oncol. 2012;7: 162. doi: 10.1186/1748-717X-7-162 23006548 PMC3504517

[17] Gunning JA, Limesand KH. Chronic phenotypes underlying radiation-induced salivary gland dysfunction. J Dent Res. 2024;103(8): 778–786. doi: 10.1177/00220345241252396 38808518 PMC11457961

[18] Functional restoration of salivary glands R01. National Institute of Dental and Craniofacial Research, National Institutes of Health. 2011. Accessed December 21, 2024. https://grants.nih.gov/grants/guide/rfafiles/rfa-de-12-004.html

[19] Functional restoration of salivary glands R21. National Institutes of Dental and Craniofacial Research, National Institutes of Health. 2011. Accessed December 21, 2024. https://grants.nih.gov/grants/guide/rfafiles/RFA-DE-12-005.html

[20] Rose SC, Larsen M, Xie Y, Sharfstein ST. Salivary gland bioengineering. Bioengineering (Basel). 2023;11(1):28. doi: 10.3390/bioengineering11010028 38247905 PMC10813147

[21] Farshidfar N, Iravani S, Varma RS. Alginate-based biomaterials in tissue engineering and regenerative medicine. Mar Drugs. 2023;21(3): 189. doi: 10.3390/md21030189 36976238 PMC10056402

[22] Hurtado A, Aljabali AAA, Mishra V, Tambuwala MM, Serrano-Aroca Á. Alginate: enhancement strategies for advanced applications. Int J Mol Sci. 2022;23(9):4486. doi: 10.3390/ijms23094486 35562876 PMC9102972

[23] Zhang Y, Pham HM, Munguia-Lopez JG, Kinsella JM, Tran SD. The optimization of a novel hydrogel-egg white-alginate for 2.5D tissue engineering of salivary spheroid-like structure. Molecules. 2020;25(23):5751. doi: 10.3390/molecules25235751 33291221 PMC7730374

[24] Li J, Sudiwala S, Berthoin L, Long-term functional regeneration of radiation-damaged salivary glands through delivery of a neurogenic hydrogel. Sci Adv. 2022;8(51):eadc8753. doi: 10.1126/sciadv.adc8753 36542703 PMC9770982

[25] Xu X, Jha AK, Harrington DA, Farach-Carson MC, Jia X. Hyaluronic acid-based hydrogels: from a natural polysaccharide to complex networks. Soft Matter. 2012;8(12):3280–3294. doi: 10.1039/C2SM06463D 22419946 PMC3299088

[26] Lee S-W, Kim J, Do M, Developmental role of hyaluronic acid and its application in salivary gland tissue engineering. Acta Biomater. 2020;115:275–287. doi: 10.1016/j.actbio.2020.08.030 32853803

[27] Young JL, Tuler J, Braden R, In vivo response to dynamic hyaluronic acid hydrogels. Acta Biomater. 2013;9(7):7151–7157. doi: 10.1016/j.actbio.2013.03.019 23523533 PMC3674107

[28] Ozdemir T, Fowler EW, Liu S, Tuning hydrogel properties to promote the assembly of salivary gland spheroids in 3D. ACS Biomater Sci Eng. 2016;2(12):2217–2230. doi: 10.1021/acsbiomaterials.6b00419 PMC5155608 27990487

[29] Srinivasan PP, Patel VN, Liu S, Primary salivary human stem/progenitor cells undergo microenvironment-driven acinar-like differentiation in hyaluronate hydrogel culture. Stem Cells Transl Med. 2017;6(1):110–120. doi: 10.5966/sctm.2016-0083 28170182 PMC5442728

[30] Yasin A, Ren Y, Li J, Sheng Y, Cao C, Zhang K. Advances in hyaluronic acid for biomedical applications. Front Bioeng Biotechnol. 2022;10:910290. doi: 10.3389/fbioe.2022.910290 35860333 PMC9289781

[31] Zhou Y, Yang Y, Liu R, Zhou Q, Lu H, Zhang W. Research progress of polydopamine hydrogel in the prevention and treatment of oral diseases. Int J Nanomedicine. 2023;18:2623–2645. doi: 10.2147/IJN.S407044 37213351 PMC10199686

[32] Snetkov P, Zakharova K, Morozkina S, Olekhnovich R, Uspenskaya M. Hyaluronic acid: the influence of molecular weight on structural, physical, physico-chemical, and degradable properties of biopolymer. Polymers. 2020;12(8):1800. doi: 10.3390/polym12081800 32796708 PMC7464276

[33] Nam K, Jones JP, Lei P, Andreadis ST, Baker OJ. Laminin-111 peptides conjugated to fibrin hydrogels promote formation of lumen containing parotid gland cell clusters. Biomacromolecules. 2016; 17(6):2293–2301. doi: 10.1021/acs.biomac.6b00588 27151393 PMC5029268

[34] Nam K, Wang CS, Maruyama CLM, Lei P, Andreadis ST, Baker OJ. L1 peptide–conjugated fibrin hydrogels promote salivary gland regeneration. J Dent Res. 2017;96(7):798–806. doi: 10.1177/0022034517695496 28208029 PMC5480806

[35] Nam K, Dos Santos HT, Maslow F, Fibrin hydrogels fortified with FGF-7/10 and laminin-1 peptides promote regeneration of irradiated salivary glands. Acta Biomater. 2023;172:147–158. doi: 10.1016/j.actbio.2023.10.013 37844750 PMC10908308

[36] Sequeira SJ, Soscia DA, Oztan B, The regulation of focal adhesion complex formation and salivary gland epithelial cell organization by nanofibrous PLGA scaffolds. Biomaterials. 2012;33(11):3175–3186. doi: 10.1016/j.biomaterials.2012.01.010 22285464 PMC3324334

[37] Xu J, Wan K, Wang H, Polyethylenimine–poly(lactic-co-glycolic acid)2 nanoparticles show an innate targeting ability to the submandibular salivary gland via the muscarinic 3 receptor. ACS Cent Sci. 2021;7(11):1938–1948. doi: 10.1021/acscentsci.1c01083 34841064 PMC8614106

[38] Cantín M, Miranda P, Suazo Galdames I, In vivo biocompatibility of the PLGA microparticles in parotid gland. Int J Clin Exp Pathol. 2013;6(11):2412–2418. 24228103 PMC3816810

[39] Muthumariappan S, Ng WC, Adine C, Localized delivery of pilocarpine to hypofunctional salivary glands through electrospun nanofiber mats: an ex vivo and in vivo study. Int J Mol Sci. 2019; 20(3):541. doi: 10.3390/ijms20030541 30696017 PMC6387464

[40] Washington MA, Balmert SC, Fedorchak MV, Little SR, Watkins SC, Meyer TY. Monomer sequence in PLGA microparticles: effects on acidic microclimates and in vivo inflammatory response. Acta Biomater. 2018;65:259–271. doi: 10.1016/j.actbio.2017.10.043 29101019

[41] Shubin AD, Felong TJ, Graunke D, Ovitt CE, Benoit DSW. Development of poly(ethylene glycol) hydrogels for salivary gland tissue engineering applications. Tissue Eng Part A. 2015;21(11–12):1733–1751. doi: 10.1089/ten.TEA.2014.0674 25762214 PMC4449707

[42] Mereness JA, Piraino L, Chen CY, Slow hydrogel matrix degradation enhances salivary gland mimetic phenotype. Acta Biomater. 2023;166:187–200. doi: 10.1016/j.actbio.2023.05.005 37150277 PMC10330445

[43] Guarnieri D, De Capua A, Ventre M, Covalently immobilized RGD gradient on PEG hydrogel scaffold influences cell migration parameters. Acta Biomater. 2010;6(7):2532–2539. doi: 10.1016/j.actbio.2009.12.050 20051270

[44] DeLong SA, Moon JJ, West JL. Covalently immobilized gradients of bFGF on hydrogel scaffolds for directed cell migration. Biomaterials. 2005;26(16):3227–3234. doi: 10.1016/j.biomaterials.2004.09.021 15603817

[45] Lin CC, Anseth KS. PEG hydrogels for the controlled release of biomolecules in regenerative medicine. Pharm Res. 2009;26(3):631–643. doi: 10.1007/s11095-008-9801-2 19089601 PMC5892412

[46] Suk JS, Xu Q, Kim N, Hanes J, Ensign LM. PEGylation as a strategy for improving nanoparticle-based drug and gene delivery. Adv Drug Deliv Rev. 2016;99(A):28–51. doi: 10.1016/j.addr.2015.09.012 26456916 PMC4798869

[47] Shubin AD, Felong TJ, Schutrum BE, Joe DSL, Ovitt CE, Benoit DSW. Encapsulation of primary salivary gland cells in enzymatically degradable poly(ethylene glycol) hydrogels promotes acinar cell characteristics. Acta Biomater. 2017;50:437–449. doi: 10.1016/j.actbio.2016.12.049 28039063 PMC5455143

[48] Ozdemir T, Fowler EW, Hao Y, Biomaterials-based strategies for salivary gland tissue regeneration. Biomater Sci. 2016;4(4):592–604. doi: 10.1039/c5bm00358j 26878077 PMC4803517

[49] Reid B, Gibson M, Singh A, PEG hydrogel degradation and the role of the surrounding tissue environment. J Tissue Eng Regen Med. 2015;9(3):315–318. doi: 10.1002/term.1688 23495204 PMC4819972

[50] Wang T, Huang Q, Rao Z, Injectable decellularized extracellular matrix hydrogel promotes salivary gland regeneration via endogenous stem cell recruitment and suppression of fibrogenesis. Acta Biomater. 2023;169:256–272. doi: 10.1016/j.actbio.2023.08.003 37557943

[51] Kort-Mascort J, Flores-Torres S, Peza-Chavez O, Decellularized ECM hydrogels: prior use considerations, applications, and opportunities in tissue engineering and biofabrication. Biomater Sci. 2023;11(2):400–431. doi: 10.1039/d2bm01273a 36484344

[52] Mushtaq F, Raza ZA, Batool SR, Preparation, properties, and applications of gelatin-based hydrogels (GHs) in the environmental, technological, and biomedical sectors. Int J Biol Macromol. 2022;218:601–633. doi: 10.1016/j.ijbiomac.2022.07.168 35902015

[53] Miyake Y, Shimizu O, Shiratsuchi H, Tamagawa T, Tonogi M. Wound healing after applying a gelatin-based hydrogel sheet to resected rat submandibular gland. J Oral Sci. 2020;62(2):222–225. doi: 10.2334/josnusd.19-0244 32224573

[54] Moroni L, Burdick JA, Highley C, Biofabrication strategies for 3D in vitro models and regenerative medicine. Nat Rev Mater. 2018;3(5):21–37. doi: 10.1038/s41578-018-0006-y 31223488 PMC6586020

[55] Shopova D, Yaneva A, Mihaylova A, Dinkova A, Bakova D. Unlocking the future: bioprinting salivary glands—from possibility to reality. J Funct Biomater. 2024;15(6):151. doi: 10.3390/jfb15060151 38921525 PMC11204800

[56] Phan TV, Oo Y, Ahmed K, Salivary gland regeneration: from salivary gland stem cells to three-dimensional bioprinting. SLAS Technol. 2023;28(3):199–209. doi: 10.1016/j.slast.2023.03.004 37019217

[57] Foraida ZI, Kamaldinov T, Nelson DA, Larsen M, Castracane J. Elastin-PLGA hybrid electrospun nanofiber scaffolds for salivary epithelial cell self-organization and polarization. Acta Biomater. 2017;62:116–127. doi: 10.1016/j.actbio.2017.08.009 28801269 PMC5646366

[58] Hong HJ, Cho J-M, Yoon Y-J, Thermoresponsive fiber-based microwells capable of formation and retrieval of salivary gland stem cell spheroids for the regeneration of irradiation-damaged salivary glands. J Tissue Eng. 2022;13:20417314221085645. doi: 10.1177/20417314221085645 PMC9003645 35422983

[59] Clyne AM. Thermal processing of tissue engineering scaffolds. J Heat Transfer. 2010;133(3):034001.

[60] Aframian DJ, Cukierman E, Nikolovski J, Mooney DJ, Yamada KM, Baum BJ. The growth and morphological behavior of salivary epithelial cells on matrix protein-coated biodegradable substrata. Tissue Eng. 2000;6(3):209–216. doi: 10.1089/10763270050044380 10941215

[61] Grenier J, Duval H, Barou F, Lv P, David B, Letourneur D. Mechanisms of pore formation in hydrogel scaffolds textured by freeze-drying. Acta Biomater. 2019;94:195–203. doi: 10.1016/j.actbio.2019.05.070 31154055

[62] Teimouri A, Azadi M, Emadi R, Lari J, Chermahini AN. Preparation, characterization, degradation and biocompatibility of different silk fibroin based composite scaffolds prepared by freeze-drying method for tissue engineering application. Polym Degrad Stab. 2015;121:18–29. doi: 10.1016/j.polymdegradstab.2015.08.004

[63] Zhang B-X, Zhang Z-L, Lin AL, Silk fibroin scaffolds promote formation of the ex vivo niche for salivary gland epithelial cell growth, matrix formation, and retention of differentiated function. Tissue Eng Part A. 2015;21(9–10):1611–1620. doi: 10.1089/ten.TEA.2014.0411 25625623 PMC4426300

[64] Lombaert IMA, Brunsting JF, Wierenga PK, Rescue of salivary gland function after stem cell transplantation in irradiated glands. PLoS One. 2008;3(4):e2063. doi: 10.1371/journal.pone.0002063 18446241 PMC2329592

[65] Nanduri LSY, Lombaert IMA, van der Zwaag M, Salisphere derived c-Kit+ cell transplantation restores tissue homeostasis in irradiated salivary gland. Radiother Oncol. 2013;108(3):458–463. doi: 10.1016/j.radonc.2013.05.020 23769181

[66] Xiao N, Lin Y, Cao H, Neurotrophic factor GDNF promotes survival of salivary stem cells. J Clin Invest. 2014;124(8):3364–3377. doi: 10.1172/JCI74096 25036711 PMC4109543

[67] Lombaert IMA, Brunsting JF, Wierenga PK, Kampinga HH, de Haan G, Coppes RP. Cytokine treatment improves parenchymal and vascular damage of salivary glands after irradiation. Clin Cancer Res. 2008;14(23):7741–7750. doi: 10.1158/1078-0432.CCR-08-1449 19047101

[68] Lin CY, Chang FH, Chen CY, Cell therapy for salivary gland regeneration. J Dent Res. 2011;90(3):341–346. doi: 10.1177/0022034510386374 21297017

[69] Lim JY, Yi T, Choi JS, Intraglandular transplantation of bone marrow-derived clonal mesenchymal stem cells for amelioration of post-irradiation salivary gland damage. Oral Oncol. 2013;49(2):136–143. doi: 10.1016/j.oraloncology.2012.08.010 22981389

[70] Mulyani SWM, Astuti ER, Wahyuni OR, Ernawati DS, Ramadhani NF. Xerostomia therapy due to ionized radiation using preconditioned bone marrow-derived mesenchymal stem cells. Eur J Dent. 2019;13(2):238–242. doi: 10.1055/s-0039-1694697 31509876 PMC6777157

[71] Carlander AF, Gundestrup AK, Jansson PM, Mesenchymal stromal/stem cell therapy improves salivary flow rate in radiation-induced salivary gland hypofunction in preclinical in vivo models: a systematic review and meta-analysis. Stem Cell Rev Rep. 2024;20(4):1078–1092. doi: 10.1007/s12015-024-10700-y 38430363 PMC11087340

[72] Lombaert I, Movahednia MM, Adine C, Ferreira JN. Concise review: salivary gland regeneration: therapeutic approaches from stem cells to tissue organoids. Stem Cells. 2017;35(1):97–105. doi: 10.1002/stem.2455 27406006 PMC6310135

[73] Blitzer GC, Rogus-Pulia NM, Mattison RJ, Marrow-derived autologous stromal cells for the restoration of salivary hypofunction (MARSH): study protocol for a phase 1 dose-escalation trial of patients with xerostomia after radiation therapy for head and neck cancer—MARSH, marrow-derived autologous stromal cells for the restoration of salivary hypofunction. Cytotherapy. 2022;24(5):534–543. doi: 10.1016/j.jcyt.2021.11.003 35183442 PMC9038658

[74] Li Z, Wang Y, Xing H, Protective efficacy of intravenous transplantation of adipose-derived stem cells for the prevention of radiation-induced salivary gland damage. Arch Oral Biol. 2015;60(10): 1488–1496. doi: 10.1016/j.archoralbio.2015.07.016 26263537

[75] Chen Y, Niu Z, Xue Y, Yuan F, Fu Y, Bai N. Improvement in the repair of defects in maxillofacial soft tissue in irradiated minipigs by a mixture of adipose-derived stem cells and platelet-rich fibrin. Br J Oral Maxillofac Surg. 2014;52(8):740–745. doi: 10.1016/j.bjoms.2014.06.006 24993354

[76] Lim JY, Ra JC, Shin IS, Systemic transplantation of human adipose tissue-derived mesenchymal stem cells for the regeneration of irradiation-induced salivary gland damage. PLoS One. 2013;8(8): e71167. doi: 10.1371/journal.pone.0071167 23951100 PMC3739795

[77] Wang Z, Ju Z, He L, Li Z, Liu Y, Liu B. Intraglandular transplantation of adipose-derived stem cells for the alleviation of irradiation-induced parotid gland damage in miniature pigs. J Oral Maxillofac Surg. 2017;75(8):1784–1790. doi: 10.1016/j.joms.2016.08.001 27621149

[78] Choi JS, An HY, Shin HS, Kim YM, Lim JY. Enhanced tissue remodelling efficacy of adipose-derived mesenchymal stem cells using injectable matrices in radiation-damaged salivary gland model. J Tissue Eng Regen Med. 2018;12(2):e695–e706. doi: 10.1002/term.2352 27860388

[79] Kojima T, Kanemaru SI, Hirano S, Regeneration of radiation damaged salivary glands with adipose-derived stromal cells. Laryngoscope. 2011;121(9):1864–1869. doi: 10.1002/lary.22080 21748735

[80] Xiong X, Shi X, Chen F. Human adipose tissue-derived stem cells alleviate radiation-induced xerostomia. Int J Mol Med. 2014; 34(3):749–755. doi: 10.3892/ijmm.2014.1837 25017690 PMC4121343

[81] Shin HS, Lee S, Kim YM, Lim JY. Hypoxia-activated adipose mesenchymal stem cells prevents irradiation-induced salivary hypofunction by enhanced paracrine effect through fibroblast growth factor 10. Stem Cells. 2018;36(7):1020–1032. doi: 10.1002/stem.2818 29569790

[82] Grønhøj C, Jensen DH, Vester-Glowinski P, Safety and efficacy of mesenchymal stem cells for radiation-induced xerostomia: a randomized, placebo-controlled phase 1/2 trial (MESRIX). Int J Radiat Oncol Biol Phys. 2018;101(3):581–592. doi: 10.1016/j.ijrobp.2018.02.034 29678523

[83] Lynggaard CD, Grønhøj C, Jensen SB, Long-term safety of treatment with autologous mesenchymal stem cells in patients with radiation-induced xerostomia: primary results of the MESRIX phase I/II randomized trial. Clin Cancer Res. 2022;28(13):2890–2897. doi: 10.1158/1078-0432.CCR-21-4520 35486613 PMC9365378

[84] Jakobsen KK, Carlander AF, Todsen T, Mesenchymal stem/stromal cell therapy for radiation-induced xerostomia in previous head and neck cancer patients: a phase II randomized, placebo-controlled trial. Clin Cancer Res. 2024;30(10):2078–2084. doi: 10.1158/1078-0432.CCR-23-3675 38441659 PMC11094414

[85] Ziebart T, Blatt S, Günther C, Significance of endothelial progenitor cells (EPC) for tumorigenesis of head and neck squamous cell carcinoma (HNSCC): possible marker of tumor progression and neovascularization? Clin Oral Investig. 2016;20(8):2293–2300. doi: 10.1007/s00784-016-1785-4 26993659

[86] Sumita Y, Iwamoto N, Seki M, Phase 1 clinical study of cell therapy with effective-mononuclear cells (E-MNC) for radiogenic xerostomia (first-in-human study) (FIH study on E-MNC therapy for radiogenic xerostomia). Med (Baltim). 2020;99(26):e20788. doi: 10.1097/MD.0000000000020788 PMC7328916 32590759

[87] Zanten JV, Jorritsma-Smit A, Westra H, Optimization of the production process of clinical-grade human salivary gland organoid-derived cell therapy for the treatment of radiation-induced xerostomia in head and neck cancer. Pharmaceutics. 2024;16(3):435. doi: 10.3390/pharmaceutics16030435 38543329 PMC10976128

[88] Ishiwata I, Tokeida Y, Iguchi M, New approach for the establishment of mouse early embryonic stem cells and induction of their differentiation. Hum Cell. 2001;14(4):283–291. 11925930

[89] Kawakami M, Ishikawa H, Tachibana T, Tanaka A, Mataga I. Functional transplantation of salivary gland cells differentiated from mouse early ES cells in vitro. Hum Cell. 2013;26(2):80–90. doi: 10.1007/s13577-013-0061-z 23681939 PMC3676639

[90] Braga MA, Tarzia O, Bergamaschi CC, Santos FA, Andrade ED, Groppo FC. Comparison of the effects of pilocarpine and cevimeline on salivary flow. Int J Dent Hyg. 2009;7(2):126–130. doi: 10.1111/j.1601-5037.2008.00326.x 19416095

[91] Porter SR, Scully C, Hegarty AM. An update of the etiology and management of xerostomia. Oral Surg Oral Med Oral Pathol Oral Radiol Endod. 2004;97(1):28–46. doi: 10.1016/j.tripleo.2003.07.010 14716254

[92] Villa A, Connell CL, Abati S. Diagnosis and management of xerostomia and hyposalivation. Ther Clin Risk Manag. 2015;11:45–51. doi: 10.2147/TCRM.S76282 25653532 PMC4278738

[93] Li KX, Loshak H. Pilocarpine for Medication-Induced Dry Mouth and Dry Eyes: A Review of Clinical Effectiveness, Cost-Effectiveness, and Guidelines. Canadian Agency for Drugs and Technologies in Health; 2019. 32129966

[94] Coppes RP, Roffel AF, Zeilstra LJ, Vissink A, Konings AW. Early radiation effects on muscarinic receptor-induced secretory responsiveness of the parotid gland in the freely moving rat. Radiat Res. 2000;153(3):339–346. doi: 10.1667/0033-7587(2000)153[0339:ereomr]2.0.co;2 10669557

[95] Teos LY, Zheng CY, Liu X, Adenovirus-mediated hAQP1 expression in irradiated mouse salivary glands causes recovery of saliva secretion by enhancing acinar cell volume decrease. Gene Ther. 2016;23(7):572–579. doi: 10.1038/gt.2016.29 26966862 PMC5798877

[96] Shan Z, Li J, Zheng C, Increased fluid secretion after adenoviral-mediated transfer of the human aquaporin-1 cDNA to irradiated miniature pig parotid glands. Mol Ther. 2005;11(3):444–451. doi: 10.1016/j.ymthe.2004.11.007 15727941

[97] Baum BJ, Alevizos I, Zheng C, Early responses to adenoviral-mediated transfer of the aquaporin-1 cDNA for radiation-induced salivary hypofunction. Proc Natl Acad Sci U S A. 2012;109(47):19403–19407. doi: 10.1073/pnas.1210662109 23129637 PMC3511089

[98] Hai B, Yan X, Voutetakis A, Long-term transduction of miniature pig parotid glands using serotype 2 adeno-associated viral vectors. J Gene Med. 2009;11(6):506–514. doi: 10.1002/jgm.1319 19326368 PMC5258189

[99] Open-label, dose-escalation study evaluating the safety of a single administration of an adeno-associated virus vector encoding human Aquaporin-1 to one parotid salivary gland in individuals with irradiation-induced parotid salivary hypofunction. ClinicalTrials.gov identifier: NCT02446249. Updated September 20, 2024. Accessed November 11, 2024. https://clinicalstudies.info.nih.gov/protocoldetails.aspx?id=15-D-0129&&query=

[100] Spirk C, Hartl S, Pritz E, Comprehensive investigation of saliva replacement liquids for the treatment of xerostomia. Int J Pharm. 2019;571:118759. doi: 10.1016/j.ijpharm.2019.118759 31622744

[101] Roblegg E, Coughran A, Sirjani D. Saliva: an all-rounder of our body. Eur J Pharm Biopharm. 2019;142:133–141. doi: 10.1016/j.ejpb.2019.06.016 31220573

[102] Llena-Puy C The role of saliva in maintaining oral health and as an aid to diagnosis. Med Oral Patol Oral Cir Bucal. 2006;11(5):E449–E455. 16878065

[103] Mystkowska J, Car H, Dąbrowski JR, Romanowska J, Klekotka M, Milewska AJ. Artificial mucin-based saliva preparations: physico-chemical and tribological properties. Oral Health Prev Dent. 2018;16(2):183–193. doi: 10.3290/j.ohpd.a40304 29736498

[104] Łysik D, Niemirowicz-Laskowska K, Bucki R, Tokajuk G, Mystkowska J. Artificial saliva: challenges and future perspectives for the treatment of xerostomia. Int J Mol Sci. 2019;20(13):3199. doi: 10.3390/ijms20133199 31261876 PMC6651665

[105] Chaudhury NM, Shirlaw P, Pramanik R, Carpenter GH, Proctor GB. Changes in saliva rheological properties and mucin glycosylation in dry mouth. J Dent Res. 2015;94(12):1660–1667. doi: 10.1177/0022034515609070 26446936

[106] Singh VK, Seed TM. The efficacy and safety of amifostine for the acute radiation syndrome. Expert Opin Drug Saf. 2019;18(11):1077–1090. doi: 10.1080/14740338.2019.1666104 31526195

[107] Varghese JJ, Schmale IL, Mickelsen D, Localized delivery of amifostine enhances salivary gland radioprotection. J Dent Res. 2018;97(11):1252–1259. doi: 10.1177/0022034518767408 29634396 PMC6151913

[108] Wie SM, Wellberg E, Karam SD, Reyland ME. Tyrosine kinase inhibitors protect the salivary gland from radiation damage by inhibiting activation of protein kinase C-δ. Mol Cancer Ther. 2017;16(9):1989–1998. doi: 10.1158/1535-7163.MCT-17-0267 28637715 PMC5587414

[109] Affandi T, Ohm AM, Gaillard D, Haas A, Reyland ME. Tyrosine kinase inhibitors protect the salivary gland from radiation damage by increasing DNA double-strand break repair. J Biol Chem. 2021;296: 100401. doi: 10.1016/j.jbc.2021.100401 33571522 PMC7973138

[110] Minagi HO, Ikai K, Araie T, Sakai M, Sakai T. Benefits of long-term pilocarpine due to increased muscarinic acetylcholine receptor 3 in salivary glands. Biochem Biophys Res Commun. 2018;503(2):1098–1102. doi: 10.1016/j.bbrc.2018.06.125 29953856

[111] Taniguchi A, Susa T, Kogo H, Iizuka-Kogo A, Yokoo S, Matsuzaki T. Long-term pilocarpine treatment improves salivary flow in irradiated mice. Acta Histochem Cytochem. 2019;52(3):45–58. doi: 10.1267/ahc.19006 31341340 PMC6643095

[112] Moral Nakamura D, da Graça Pinto H, Baena Elchin C, Efficacy of bethanechol chloride in the treatment of radiation-induced xerostomia in patients with head and neck cancer: a systematic review and meta-analysis. Radiother Oncol. 2023;186:109715. doi: 10.1016/j.radonc.2023.109715 37207874

[113] Yang WF, Liao GQ, Hakim SG, Ouyang DQ, Ringash J, Su YX. Is pilocarpine effective in preventing radiation-induced xerostomia? A systematic review and meta-analysis. Int J Radiat Oncol Biol Phys. 2016;94(3):503–511. doi: 10.1016/j.ijrobp.2015.11.012 26867879

[114] Jeong YJ, Park SH, Mun SH, Kwak SG, Lee SJ, Oh HK. Association between lysyl oxidase and fibrotic focus in relation with inflammation in breast cancer. Oncol Lett. 2018;15(2):2431–2440. doi: 10.3892/ol.2017.7617 29434955 PMC5777281

[115] Yang N, Cao D-F, Yin X-X, Zhou H-H, Mao X-Y. Lysyl oxidases: emerging biomarkers and therapeutic targets for various diseases. Biomed Pharmacother. 2020;131:110791. doi: 10.1016/j.biopha.2020.110791 33152948

[116] Muir AJ, Levy C, Janssen HLA, ; for the GS-US-321–0102 Investigators. Simtuzumab for primary sclerosing cholangitis: phase 2 study results with insights on the natural history of the disease. Hepatology. 2019;69(2):684–698. doi: 10.1002/hep.30237 30153359

[117] Shanbhag V, Jasmer-McDonald K, Zhu S, ATP7A delivers copper to the lysyl oxidase family of enzymes and promotes tumorigenesis and metastasis. Proc Natl Acad Sci U S A. 2019;116(14):6836–6841. doi: 10.1073/pnas.1817473116 30890638 PMC6452744

[118] Nam K, Dos Santos HT, Maslow FM, Copper chelation reduces early collagen deposition and preserves saliva secretion in irradiated salivary glands. Heliyon. 2024;10(2):e24368. doi: 10.1016/j.heliyon.2024.e24368 38298614 PMC10828693

